# Awareness and practices of contraceptive use among university students in Botswana

**DOI:** 10.1080/17290376.2013.869649

**Published:** 2014-01-03

**Authors:** M.E. Hoque, T. Ntsipe, M. Mokgatle-Nthabu

**Affiliations:** a MSc, is affiliated to Graduate School of Business and Leadership at the University of KwaZulu-Natal, Westville Campus, South Africa; b MPH, is affiliated to Department of Public Health at the University of Limpopo (Medunsa Campus), South Africa; c PhD, is affiliated to Department of Public Health at the University of Limpopo (Medunsa Campus), South Africa

**Keywords:** awareness, utilization, contraception, university students, Botswana, conciencia, la utilizatión, la anticoncepción, los estudiantes universitarios, Botswana

## Abstract

In Botswana, unplanned pregnancies, especially among the youth constitutes a growing health and social problem. Research in the field of contraceptive practices, and the causes of sexual practices in Botswana, remains scarce and relatively limited. The objectives of this study was to investigate the awareness and utilization of various contraceptive methods, among university students in Botswana. A descriptive, cross-sectional, research study was conducted among 346 randomly selected students, who completed confidential, self-administered questionnaires. The average age of the respondents was 21 years (SD = 2.8 years). The level of awareness among students regarding contraception was good (score ≥9). Both the male and the female students had almost similar awareness level of contraceptive use, as their mean scores were 8.79 and 8.72, respectively (*p* = .733). All the female students (100%) were ‘aware’ that the effectiveness of the contraceptives used, as compared to male students, being 93.7%. A greater proportion of the female students (90.6%) knew that using contraceptives irregularly would result in pregnancy, in contrast to 76.4% males. More than half (59.0%) of the students indicated that they had engaged in sexual acts. Significantly, more male students (68.5%) had sexual experiences prior to the study, compared to 54.5% of their female counterparts (*p* = .038). The majority of the students (76%) reported that they had always used contraceptive methods. The most commonly used contraceptive method was the condom (95.6%), followed by oral contraceptive pill (86.7%). There was no significant association found between the level of awareness and the use of contraceptives. Results suggested that many students still engaged in risky, contraceptive practices by engaging in unprotected sexual acts. Therefore, there is a need to educate the students about sexually transmitted infections, the different contraceptive methods and the regular use of the available contraceptives.

## Abstract (FR)

Au Botswana, les grossesses non désirées, en particulier parmi les jeunes constitue un problème croissant et la santé sociale. La recherche dans le domaine des pratiques contraceptives, et les causes de pratiques sexuelles au Botswana, demeure rare et relativement limitée. Les objectifs de cette étude, était d'enquêter sur la sensibilisation et l'utilisation de diverses méthodes de contraception, parmi les étudiants de l'université du Botswana. Une coupe transversale, étude descriptive, la recherche a été menée auprès de 346 étudiants choisis au hasard, qui ont terminé, les questionnaires auto – administrés confidentielles. L'âge moyen des répondants était de 21 ans (SD=2,8 années). Le niveau de sensibilisation des étudiants concernant la contraception était bon (score ≥9). Deux des étudiants les hommes et les femmes avaient le niveau de sensibilisation è peu près semblable de l'utilisation des contraceptifs, comme leurs scores moyens étaient de 8,79 et 8,72 respectivement (p=0,733). Tous les éleèves de sexe féminin (100%) ont été «conscient» que l'efficacité des contraceptifs utilisés, par rapport aux garçons, étant de 93,7%. Une plus grande proportion des élèves de sexe féminin (90,6%) savait que l'utilisation de contraceptifs irrégulieère serait aboutir à une grossesse, contrairement à 76,4 % des hommes. Plus de la moitié (59,0%) des élèves ont indiqué qu'ils avaient participé à des actes sexuels. De manière significative, plus de garçons (68,5%) ont eu des expériences sexuelles avant l'étude, comparativement à 54,5% de leurs homologues féminins (p=0,038). La majorité des étudiants (76.0%) ont déclaré qu'ils avaient toujours utilisé des méthodes contraceptives. La méthode de contraception la plus utilisée était le préservatif (95,6%), suivie par la pilule contraceptive (86,7%). Il n'y avait pas d'association significative entre le niveau de connaissance et l'utilisation de contraceptifs. Les résultats suggèrent que de nombreux étudiants encore engagés dans les pratiques contraceptives, risque en s'engageant dans des actes sexuels non protégés. Par conséquent, il est nécessaire d'éduquer les étudiants sur les sexuellement transmissibles infections (STI's), les différentes méthodes de contraception et l'utilisation régulière de contraceptifs disponibles.

## Introduction

An unplanned or an unwanted pregnancy is a serious, global public health problem (Kang & Moneyham 2008). It is estimated that about 80 million, unplanned pregnancies occurs in the world every year (Speidel, Harper & Shields 2008). Unplanned pregnancies may be prevented by using the contraceptive methods, such as the oral contraceptive pills, the long-term hormonal injections, the condoms, the tubal ligation or a vasectomy (Contraceptive Chart 2008). There are also emergency contraceptives available to prevent unplanned pregnancies, and should be obtained and taken within 72 hours after engaging in unprotected sexual intercourse (Steyn & Mason 2009).

Contraceptives are available with no charge from the government health facilities in Botswana. These facilities include referral hospitals, primary hospitals, main clinics, university clinics, mobile clinics, health posts and the Botswana Family Welfare Association, and sexual and reproductive centres. These health centres are located within a 15-kilometre radius from the residents homes (UNFPA Case Study 2003). In Botswana, a study done on the youth on reproductive and sexual health issues, revealed that over a quarter of the women aged 15–19 years were pregnant (Advocates for Youth 2009). The overall prevalence rate of contraceptive use among married women was 44% (Population Reference Bureau 2012). Another study found that one in every three women who were single or teenagers and between the ages of 15 and 49 years was pregnant because of unprotected sex (Mookodi, Ntshebe & Taylor 2004).

In Botswana, about 90% of the university student's fees are sponsored by the government. These university students are classified within the university community as ‘unplanned pregnancy groups', and viewed as being ‘not yet ready to be parents'. The unplanned pregnancies may occur as a result of not utilizing contraceptive methods or due to the failure of the chosen contraceptive methods used. Therefore, the unplanned pregnancies that occurs each year has a negative impact on Botswana's economy. It impacts on the tax payers while the students may extend and interrupt their studies. While contraceptives methods are offered free of charge in government health facilities, the information on contraceptive use in general, is not easily available in Botswana to the young students (Advocates for Youth 2009). Therefore, the objectives of this study were to investigate the awareness and practices of contraception among the University students in Botswana.

## Methodology

### Study design and setting

This study used a descriptive, cross-sectional study design. The setting was the main campus at the University of Botswana in Gaborone, Botswana. This University was established in 1982 and has four campuses, two in Gaborone, one in Francistown and one in Maun. The seven specialities offered within the faculties are Business, Education, Engineering, Humanities, Science, Social Sciences and the School of Medicine.

### Study population

The study population consisted of all the registered, undergraduate students ranging from the first to the fifth years at the University of Botswana, main campus in Gaborone. Currently there are 14,093 students, 11,587 are full-time, 2614 part-time and 505 distance learning students. The total enrolment in the faculties are 26% in business, 12% education, 6% engineering, 3% medicine, 17% humanities, 9% science and 16% social sciences. Approximately 45% of the students' were males and 55% were females.

### Sample size

A minimum sample size was used for the study, and was calculated, using a standard formula as described by Reid and Boore (1991). The total population of students at the university was 14,093. A sample size of 389 was obtained. Ten per cent more of the sample was added to the total to cover the number of dropouts of the students. Therefore the calculated sample size was 428.

### Inclusion and exclusion criterion

The respondents were all the registered, full-time undergraduate students from all years of study at the main campus of the University of Botswana. Both males and females, pregnant or non-pregnant that were willing to participate in the study were included in the study. All part-time undergraduates and postgraduates (part time or full time) students from the main campus of the University of Botswana were excluded from the study.

### Ethical considerations

Ethical approval for the study was obtained from the University of Limpopo, Medunsa Research and Ethics Committee, South Africa, the Human Research Development Committee of the Ministry of Health Botswana, and the Director of Student Affairs, University of Botswana, Gaborone Botswana. The informed consent form was signed and obtained from the respondents. Confidentiality of the respondents was maintained at all times. Anonymity was maintained by not disclosing the identity of the respondents. Participation was voluntary and the respondents were informed that they could withdraw from the study at any stage, if they so desired without any penalty.

### The instrument

The questionnaire had three sections. The first section consisted of demographic information such as age, sex, year of study, etc. The second section consisted of ‘awareness’ regarding questions on contraception. This section had 14 questions of which 13 questions had yes, no and do not know answers and only one was correct. The last question consisted of multiple answers requesting the respondents to indicate the methods of contraception. The last section dealt with respondent's utilization of contraceptives.

### Data collection

Simple random sampling method was used to select samples for the study. The students were recruited from strategic points and areas used by the students within the university community. The strategic points selected were the University Library entrance, the Dining Area (Refectory), the Tuck-shop and the student's community centre. A self-administered questionnaire was used to collect the data and was distributed from the main campus. The questionnaires were in English as this is the medium used to teach at the University of Botswana.

Pre-testing of the questionnaire was conducted using 20 students from the college of health sciences to identify gaps and modify the questionnaire. The students who participated in the pre-test were not part of the main study.

### Data analysis

Data were entered into a Microsoft Excel 2003 spreadsheet and analysed, using the SPSS 18.0.1. The demographics and outcome variables were summarized using descriptive summary measures, expressed as means (SD) for continuous variables and percentage for categorical variables. Chi-square test was used to determine the test of association between the categorical variables. All the statistical tests were performed using two-sided tests at the .05 level of significance. *P*-values reported to three decimal places with values less than .001 reported as <.001. *P*-values less than .05 were considered statistically significant.

## Results

A total of 346 students completed the questionnaires, with a response rate of 80.8%. Table 1 shows the demographic information of the respondents. The mean age of the students was 20.51 years (SD = 2.85 years). The majority of the students (75.5%) were between the ages 19 – 23 years and 68% were females. More than a third of the students were from first year (38.4%) and second years (35.5%), respectively. The majority of the respondents (82.4%) stayed off-campus, and among them 76.5% stayed with their parents and 16.1% staying alone.

**Table 1.** Demographic information of the students (*n* = 346).

Variables	Frequency	Percentage
*Age of the undergraduate students*		
≤ 18 years	53	15.3
19–23 years	261	75.4
≥ 24 years	32	9.2
Mean age (SD)	20.51 years (SD = 2.85)	
*Year of undergraduate study*		
1st year	133	38.4
2nd year	123	35.5
3rd year	43	12.4
4th year	44	12.7
5th year	3	0.9
Hostel residence on campus	61	17.6
Accommodation off–campus	285	82.4
*Accommodation off-campus shared with:*		
Parents	218	76.6
Alone	46	16.1
Partner	19	6.7
Spouse	2	0.7

### Awareness regarding contraceptives

Overall, the male and female students had ‘good awareness regarding contraceptives’ as more than half of them (58.6% and 59.1%) for both males and females respectively scored nine or above. The males and the females also had almost ‘similar awareness’ as their mean scores were 8.79 and 8.72, respectively (*p* = .733). The respondents’ responses regarding contraception are summarized in Table 2. All the female students (100%) were ‘aware’ that contraceptives are not 100% effective as compared to 93.7% of the male students. More females (90.6%) compared to males (76.4%) knew that using contraceptives irregularly will result in pregnancy. In terms of the other variables, there was no variation noted between the ‘awareness’ of females as compared to the awareness of male. The most commonly known contraceptive method used was the condom (95.6%) followed by 86.7% of the oral contraceptive pill.

**Table 2.** Awareness regarding contraception of the students by gender (%)

Awareness statements	Male (*n* = 111)	Female (*n* = 235)
Contraceptives are free of charge in Botswana (true)	76.6	80.9
Natural family planning methods (withdrawal)	62.2	70.2
Contraception can only be used by married people (false)	95.5	97.4
Contraception can result in pregnancy (false)	42.3	43.0
Contraception should be used all the time as long as sexually active (true)	76.6	82.6
After unprotected sex, not necessary to continue (false)	80.2	80.0
90% of women who use contraceptives do not experience, unplanned pregnancy (true)	59.5	56.6
Contraception is 100% effective (false)	93.7	100.0
Irregular use of contraception will not result in pregnancy (false)	76.4	90.6
Prolonged use of contraception results in sterility (false)	45.9	39.6
One type, should not be given any information (false)	86.4	90.6
Default. cannot fall pregnant (false)	66.6	63.8
Unprotected sex at least once cannot fall pregnant (false)	86.5	90.2
All contraceptives contains hormones (false)	50.5	54.0
*Contraceptive methods*		
Condom	94.6	96.6
Pill	81.1	89.4
Loop	65.8	77.4
Injection	57.7	74.0
Natural methods	31.5	38.3
Norplant	19.8	26.8

Table 3 shows the respondents contraceptive practices. More than half (59.0%) of the students indicated that they had engaged in sexual intercourse. More male students (68.5%) had sex before the study as compared to their female counterparts (54.5%), and the rates were significantly different (*p* = .038).

**Table 3.** Contraceptive practices among students (%).

Contraceptive practices	Male (*n* = 111)	Female (*n* = 235)
Had sex before (yes)	68.5	54.5
*Among sexually active students, how often use contraception*^a^		
Always	80.3	73.4
Sometimes/never	19.7	26.4
*Contraceptive method used*^b^		
Condom	97.1	97.8
Natural method	15.9	11.2
Pill	4.3	11.2
Loop	4.3	0.8
Injection	2.9	0.8
*What one can do after unprotected sex*		
I will wash myself	1.6	2.4
Visit the nearest clinic	42.2	49.2
Wait for a month to see if pregnant	10.9	11.9
Seek emergency pill	33.8	33.9
Do not know	19.7	13.1

a Use of more than one method of contraceptive.

b Males *n* = 76, females *n* = 128.

The main source of information received regarding contraceptives use was the school or the health facility (78.3%) followed by television and radio (Table 4). Students reported that their preferred places of accessing contraceptives were the clinics followed by the university clinics. No significant association was found between the ‘awareness level’ and the use of contraceptives (*p* = .085).

**Table 4.** Students' source of information and access point concerning contraceptives (%).

Sources of information	Male (*n* = 111)	Female (*n* = 235)
School/health facilities	73.9	80.4
Television	64.8	68.1
Radio	57.6	58.7
Printed material/billboards/magazine	50.5	60.0
Parents	27.9	36.2
Internet	1.8	3.8
Peers	0.0	3.0
*Preferred point for accessing contraceptives*		
Clinics	43.2	32.3
University clinic	37.8	23.0
Pharmacy	27.7	21.7
Hospital	22.5	21.3
Youth facility	18.0	14.0
Supermarket, shop, bar	9.0	7.7

## Discussion

This study aimed to investigate university students awareness and practices regarding contraceptive use in Botswana. Overall, the students had an above average awareness level and the majority used some form of contraceptive. No association was found between the awareness level and contraceptive practices.

The mean age of the students was 21 years (± 2.85) and most of the students were between the ages of 19 and 23 years. In Botswana, 80% of the children start school at around seven to eight years of age; therefore most students were within this age group, as they were now 19 years and since completion of secondary school. More than 60% were females and this was quite similar to the University of Botswana student proportion by gender 2009/2010, as there were more females (55%) as compared to males (45%) (University of Botswana 2010).

### Awareness regarding contraceptives

The respondents in this study generally had good awareness regarding contraceptives. All the female students knew that contraception was not 100% effective. Irregular uses mean using contraceptives sometimes, for example using condom sometimes, skipping the pill or delayed taking of the injectable after the scheduled type. The findings of this study are contrary to the findings of a study in South Africa, as it was found that awareness regarding irregular use was 21% among female respondents as compared to 90.6% among female respondents in this study (Oni, Prinsloo, Nortje & Joubert 2005). The condom (95.6%) was the most commonly known method followed by the oral contraceptive pill (86.7%). This finding contradicted earlier findings of Mookodi *et al.* (2004), where the condom was reported to be known to all the participants. In a South African study, awareness of contraception on condom use among the males and females was 72.9% and 74.2% (Oni *et al.* 2005). Norplant was the least known method (24.6%). But this finding is much higher than the Nigerian study, where only 5.7% students were aware of Norplant (Oyedokun 2007).

About two-thirds of the respondents mentioned that they had engaged in sexual intercourse. This is similar to the South African study which reported that 65.7% of the university students had had sex prior to the study (Hoque & Ghuman 2011). Other studies from Africa reported higher rates of having sex among the university students, namely, 70% in Uganda, and 80% from Madagascar, had sex prior to the study (Rahamefy, Rivard, Ravaoarinoro, Ranaivoharisoa, Rasamindrakotroka & Morisset 2008; Sekirime, Tamale, Lule & Wabwire-Mangen 2001). A Nigerian study reported that 54% undergraduate students were sexually active (Omoteso 2006).

The majority of the sexually active respondents indicated that they were using contraceptives, even though a quarter were not using them regularly. Studies from Ethiopia and Nigeria concluded that only 10% and 30.1% used contraceptives regularly (Oyedokun 2007; Tamire & Enqueselassie 2007). A recent South African study reported that 67.8% of the sexually active students were using contraceptives, sometimes or rarely (Hoque & Ghuman 2011).

The findings of this study showed that the condom is the most commonly used method, among both males and females, followed by the withdrawal method. The finding of this study is similar with that of the Central Statistics Office (2006) which reported that 93.3% of the sexually participants used condoms. The study in Ethiopia reported that the contraceptive method mostly used was the pill, followed by the injection (Tamire & Enqueselassie 2007). Botswana is one of the countries most severely affected by HIV/AIDS. Condoms were highly marketed and available almost everywhere in the country.

The present study found that both males and females (61.1%) preferred to access contraceptives from clinics, but not the university clinic. The university clinic was the next preferred followed by pharmacies. In Botswana as contraceptives could be accessed free of charge in government facilities, it is not surprising to see the clinics being the main access points, but the same was expected for hospitals and youth facilities as well. One may argue that the clinics are many and easily accessible, as compared to the hospitals; however, the youth facilities are also many but different trends on preferences was found. The reason may be due to the lack of information about the youth facilities, unfriendly attitudes of service providers to youth or inconvenient hours as was mentioned in a case study by UNFPA Case Study (2003).

The findings of this study indicated that the most common source of information obtained regarding contraception, was mostly from schools and health facilities. This is contrary to a previous study from Botswana which found that the most common source was friends and HIV programmes (Francoeur & Noonan 2004). This is also in contrast to a study done in Nigeria whereby 51.2% indicated hospital or clinics (Oyedokun 2007). Parents giving information on contraceptives was among the least, and this supports literature as the findings revealed that parents in Botswana are not comfortable talking about sexuality with their children (Advocates for Youth 2009).

No association was found between the level of awareness and contraceptive use. The awareness level was ‘good but’ there is still a need to investigate, if this ‘awareness results’ can be acceptable for use, since literature generally agrees that better ‘awareness’ about contraception, increases the chances of better contraceptive practices (Oyedokun 2007).

## Limitations of study

There were numerous limitations that could arise from the study methodology. The study population consisted of students at one university, and it might not be advisable to generalize our results with regard to other universities. Students' behaviours were self-reported. Although the surveys were anonymous, there might be information bias because some students may have been reluctant to report sensitive information regarding contraceptive practices. To minimize this type of bias, confidentiality was ensured.

## Conclusion

The study found that the level of ‘awareness’ was ‘good’ between both males and females and was ‘satisfactory’ to sustain adequate contraceptive use, in the context of the ‘high’ unplanned pregnancy rate. The condom was the most popular method used by most respondents, showing that the HIV/AIDS prevention strategy in Botswana is working well, in attracting condom use by this student population. Sexual practices of some of the respondents remain at risk, as there are still many students engaging in unprotected sexual acts. Contraceptive use has to be ‘high’, even though there were instances of infrequent use, with the most commonly used method being the condom. The university management should organize workshops on regular basis at the university on changing reproductive health and sexual behaviour, as well as health education programmes.
